# Fibroblast growth factor-2 stimulates proliferation of human adipose-derived stem cells via Src activation

**DOI:** 10.1186/s13287-019-1462-z

**Published:** 2019-11-27

**Authors:** Yuanyuan Ma, Natsuko Kakudo, Naoki Morimoto, Fangyuan Lai, Shigeru Taketani, Kenji Kusumoto

**Affiliations:** 1 Department of Plastic and Reconstructive Surgery, Kansai Medical University, 2-5-1 Shin-machi, Hirakata, Osaka, 573-1010 Japan; 2 International Joint Research Laboratory for Cell Medical Engineering of Henan, Kaifeng, Henan 475-000 China; 3 Department of Plastic and Reconstructive Surgery, Huaihe Hospital of Henan University, Kaifen, Henan 475-000 Japan; 4 Department of Plastic and Reconstructive Surgery, Kyoto University, Kyoto, 606-8501 Japan; 5 Department of Microbiology, Kansai Medical University, Osaka, 573-1010 Japan

**Keywords:** Fibroblast growth factor-2, Human adipose-derived stem cells, Stem cell proliferation, Src activation, Signaling pathway

## Abstract

**Background:**

Human adipose-derived stem cells (hASCs) are a subset of mesenchymal stem cells (MSCs); it has been regarded as one of the most promising stem cells. We previously found that fibroblast growth factor-2 (FGF-2) enhanced the proliferation and differentiation of hASC. However, the mechanisms involved in the growth of hASCs by FGF-2 have not been investigated.

**Methods:**

Human adipose-derived stem cells (hASCs) were cultured with FGF-2, and cell growth was assessed. Effects of FGF Receptor (FGFR) inhibitor (NVP-BGJ398), ERK1/2 inhibitor (PD98059), PI3K/Akt inhibitor (LY294002), JNK inhibitor (SP600125), and p38 MAPK inhibitor (SB203580) and Src inhibitor (PP1) on the proliferation were investigated. At the same time, we assessed the effect of FGFR inhibitor on several signaling enzymes such as ERK1/2, JNK, p38, and Akt, in protein level. The involvement of Src activation by FGF-2 was also examined.

**Results:**

FGF-2 markedly promoted proliferation of hASCs at concentrations lower than 10 ng/ml and stimulated cell progression to the S and G2/M phases. Proliferation was blocked by the FGFR inhibitor (NVP-BGJ398) and various signaling pathway inhibitors, such as Erk1/2 inhibitor (PD98059), PI3K/Akt inhibitor (LY294002), JNK inhibitor (SP600125), and p38MAPK inhibitor (SB203580). The FGFR inhibitor reduced the activation of protein kinases, such as AKT, Erk1/2, JNK, and p38, in several signaling pathways. The downstream kinase of FGFR, Src, was activated by FGF-2, and its activation was canceled by the FGFR inhibitor. MEK1/2, a downstream kinase of Src, was parallelly regulated by FGF-2. The Src inhibitor (PP1) markedly blocked the proliferation of hASCs via inhibition of Src and MEK1/2.

**Conclusion:**

Src activation is indispensable for FGF-2-mediated proliferation of ASCs, as well as the subsequent activation of multi-signaling pathways.

## Introduction

Human adipose-derived stem cells (hASCs) were initially identified and described as a putative population of multipotent stem cells [1]. hASCs have been one of the most promising stem cells [2], the advantages of which are as follows: (1) hASCs can be ubiquitous and easily harvested in larger quantities without significant donor-site morbidity [3, 4]; (2) later senescence [5, 6], higher proliferation capacity [7], and more active in autocrine production of kinds of bioactive factors as well as extracellular vesicles [8, 9]; (3) less ethical and legal issues compared with embryonic stem cells as they can be used in the autologous form; additionally, they lack the ability to express MHC-class II antigens, express low levels of MHC-class I antigens, and have less mutational and other adverse effects compared with induced pluripotent stem cells (iPSCs) [8, 10]; and (4) hASCs can be cryopreserved for a long time [11]. As for the advantages of hASCs, there is increasing interest in advanced tissue engineering and cell therapies in scientific and clinical research [8, 12–14].

Fibroblast growth factor-2 (FGF-2) is one member of the FGF family, which has multiple functions such as mitogenesis, migration, morphogenesis, angiogenesis, organ development, organ regeneration, and wound healing [15]. Recent studies have shown the proliferation and differentiation effects of FGF-2 on different original stem cells [16–18] and the proliferation effect on hASCs [19–21]. Zaragosi et al. reported that FGF-2 plays a crucial role in differentiation and proliferation of hASCs, and that the Erk1/2 pathway is involved [20]. Our previous study found that FGF-2 increased hASC proliferation [22]; however, the signaling pathways activated by FGF-2 remain unexplored. For better use of hASCs in clinical therapy, it is necessary to understand the activation mechanism of hASC proliferation.

Src is a Src kinase family member that is anchored to the cytoplasmic side of the cell membrane and is important in the regulation of growth and differentiation of eukaryotic cells [23]. Src can also alter the renewal and repopulation of hematopoietic stem cells [24, 25], the proliferation and differentiation of mouse spermatogonial stem cells [26, 27], and the differentiation of human pluripotent stem cells and embryonic stem cells [28, 29]. However, the influence of Src on hASCs is still unknown.

Thus, the aim of the present study was to investigate whether FGF-2 enhances the proliferation of hASCs, including investigation of the following: (1) the proliferation effect of hASCs in the appearance of FGF-2 in which signaling pathways are involved in FGF-2-mediated proliferation and (2) the effect of a Src inhibitor on FGF-2-mediated proliferation. Here, we demonstrated the requirement of Src activation for FGF-2-mediated proliferation.

## Materials and methods

### Reagents and antibodies

Fibroblast growth factor-2 was from Kaken Pharmaceutical (Tokyo, Japan). Fetal bovine serum (FBS) was from Hyclone (Logan, UT, USA). PD98059 (MEK1/2 inhibitor), LY294002 (phosphatidylinositol-3-kinase-Akt inhibitor), and SP600125 (JNK inhibitor) were from Selleck Biotech (Osaka, Japan); SB203580 (p38 MAPK inhibitor) was from Sigma (Merck KGaA, Darmstadt, Germany); NVP-BGJ398 (FGFR inhibitor) was from Santa Cruz Biotechnology (Tokyo, Japan); and PP1 (Src inhibitor) was from Cayman (Cayman Chemical, USA). Mouse anti-SAPK/JNK, rabbit anti-phospho-p38 MAPK, rabbit anti-phospho-Src family, rabbit anti-Phospho-MEK1/2, rabbit anti-phospho- p44/42 MAPK (Erk1/2), and rabbit anti-β-actin were from Cell Signaling Technology (Beverly, MA, USA); rabbit anti-phospho-Akt and rabbit anti-phospho-Akt (Ser473) (D9E) was from Abcam (Cambridge, UK); and peroxidase-linked secondary antibody was from GE Healthcare (Little Chalfont, UK).

### Isolation of human adipose-derived stem cells (hASCs)

Human adipose tissue was harvested from the abdominal subcutis of patients who underwent plastic surgery. hASCs were isolated as previously described [30, 31]. One-third volumes of adipose tissue were rinsed three times with equal volumes of phosphate-buffered saline (PBS), minced into small pieces, and digested with 0.1% type II collagenase solution (Sigma-Aldrich, St. Louis, MO), shaking at 40 °C for 40 min. After digestion, Dulbecco’s modified Eagle’s medium (DMEM) containing 10% fetal bovine serum (FBS) and 2% penicillin/streptomycin (P/S, Gibco, Tokyo, Japan) (complete medium) was added to the same volume of adipose tissue, then centrifuged at 1600 rpm for 3 min. The floating population of mature adipocytes was discarded, and the stromal vascular fraction (SVF) was rinsed three times with PBS before filtering with a 100-mm nylon mesh (BD Falcon, Bedford, MA, USA). Then, cells were incubated in complete medium at 37 °C in a humidified air atmosphere with 5% CO2. The adhered hASCs were changed into fresh complete medium every 2 days and reached 80–90% confluence after 6–7 days. Cells were passaged using TrypLE Express (Gibco, Grand Island, USA), and nearly all cells formed fibroblast-like cells after 3 passages in complete medium. The fibroblast-like hASCs were frozen in an alcohol freezing container and stored in a liquid N_2_ tank. For all experiments, cells from passages 7–9 were used. All subjects enrolled in this research provided informed consent, and the study protocol was approved by the Institutional Committee on Human Research of Kansai Medical University.

### Cell proliferation assay

hASCs were seeded into 24-well cell culture plates at a density of 1.0 × 10^4^ cells/well and incubated in complete medium overnight. The cell medium was then replaced with serum-free DMEM containing 0.1% FBS and 0.2% streptomycin (control medium). After culturing for 18 h, hASCs were treated with FGF-2 (Kaken Pharmaceutical, Tokyo, Japan) at the stated concentrations in serum-free DMEM for 48 h. Inhibitors included the MEK1/2 inhibitor PD98059 (Selleck Biotech, Osaka, Japan), the phosphatidylinositol-3-kinase-Akt inhibitor LY294002, the p38 MAPK inhibitor SB203580, the JNK inhibitor SP600125, the FGFR inhibitor NVP-BGJ398 and the Src inhibitor PP1. Inhibitors were added 1 h prior to incubation in FGF-2. Cell proliferation was tested by Cell Counting Kit-8 (Dojindo Molecular Technologies, Kumamoto, Japan) according to the manufacturer’s instructions. Absorbance was read at 450 nm on a multi-well plate reader (EnSpire 2300 Multilabel Reader; PerkinElmer, Inc., Waltham, MA, USA).

### Cell cycle assay

hASCs (3 × 10^5^ cells) were seeded into 10-cm diameter cell culture dishes containing complete medium and cultured overnight. The medium was then replaced with control medium for 18 h. After adding inhibitor (NVP-BGJ398) for 1 h, reagents (FGF-2 +/− NVP-BGJ398) were added into the dishes at the stated concentrations and culturing for 48 h. Cells were transferred from dishes to 15-ml tubes using TrypLE Express. After washing twice with ice-cold PBS, cells were fixed in 70% ethanol at − 20 °C for at least 3 h. The fixed cells were then stained with Muse™ Cell Cycle kit (Millipore, Merck KGaA, Darmstadt, Germany) in the dark at room temperature for 30 min. Cell cycle phases were analyzed by flow cytometric quantification of DNA with the Muse™ Cell Analyzer (Millipore).

### Western blot analysis

hASCs (4 × 10^5^ cells) were seeded into 10-cm cell culture dishes containing complete medium and cultured overnight. The medium was then replaced with control medium for 18 h. After adding inhibitors (NVP-BGJ398/PP1) for 1 h, reagents (FGF-2 +/− NVP-BGJ398, FGF-2+/−PP1) were added into the dishes at the stated concentrations and culturing for 5 min. The cells were lysed in a mixed solution of M-PER (mammalian protein extraction reagent, Thermo): phosphatase inhibitor cocktail (Nacalai tesque, Kyoto, Japan) =100:1. Extracted cellular proteins (20 μg) were separated by sodium dodecyl sulfate-polyacrylamide gel electrophoresis (SDS-PAGE) and then transferred to a polyvinylidene difluoride (PVDF) membrane (Invitrogen, USA). The membrane was first blocked with Blocking One-P reagent (Nacalai Tesque, Kyoto, Japan) for 60 min at room temperature and then incubated with the following primary antibodies: rabbit anti-phospho-p44/42 MAPK (Erk1/2) (Thr202/Tyr204) (1:1000), rabbit anti-phospho-Akt, rabbit anti- phospho-Akt (Ser473) (D9E) (1:1000), mouse anti-SAPK/JNK (Thr183/Tyr185)(G9) (1:1000), rabbit anti-phospho-p38 MAPK (Thr180/Tyr182)(D3F9) (1:1000), rabbit anti-β-actin (13E5) (1:1000), rabbit anti-phospho-Src family (Tyr416) (1:1000), and rabbit anti-Phospho-MEK1/2(Ser217/221) (41G9) (1:1000) at 4 °C overnight. This was followed by incubation with peroxidase-linked secondary antibody (1:20,000) at room temperature for 60 min. The labeled proteins were detected with enhanced chemiluminescence using the Prime Western blotting detection system (GE Healthcare).

### Statistical analysis

Statistical analyses were performed using Microsoft Excel (Office 2013; Microsoft Corporation, Redmond, WA, USA) and Prism 7.03 (GraphPad Software, Inc., San Diego, CA, USA) software programs. The Mann–Whitney *U* test was used to evaluate differences among groups. All data are presented as the mean ± standard error of mean (SEM). *p* < 0.05 was considered statistically significant.

## Results

### FGF-2-mediated proliferation of hASCs

**Fig. 1:**
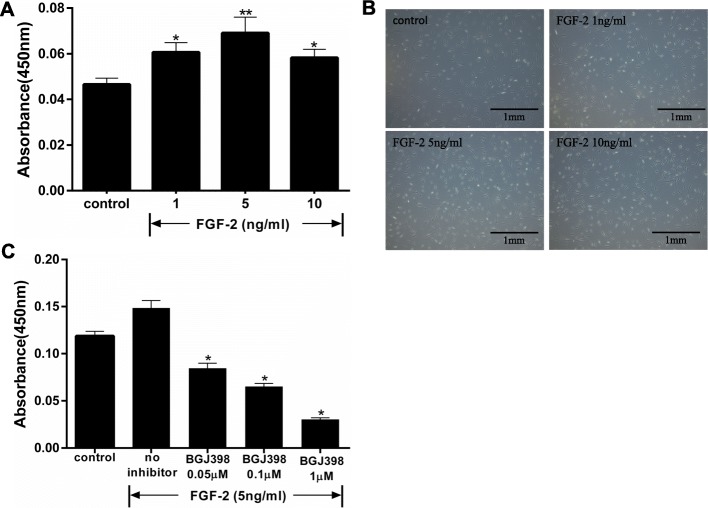
Effect of different concentrations of FGF-2 on hASCs proliferation. Cells were incubated with FGF-2 in serum-free DMEM for 48 h. Growth was examined with a Cell Counting Kit-8 by reading absorbance at 450 nm. **a** FGF-2 stimulated hASC proliferation (*n* = 8) in a concentration-dependent manner. *p < 0.05 and **p < 0.01 vs controls. **b** Phase-contrast micrographs show an increase in hASCs after treatment with FGF-2. **c** Effect of NVP-BGJ398 (FGFR inhibitor) on FGF-2-mediated proliferation of hASCs (*n* = 5). *p < 0.01 compared with no inhibitor

Proliferation of hASCs was increased by treatment with 1 ng/ml FGF-2 (0.01 < *p* < 0.05 vs control), and 5 ng/ml FGF-2 stimulated cell proliferation to a greater extent (*p* < 0.01 vs control). Thus, FGF-2 stimulated proliferation of hASCs in a dose-dependent manner up to 10 ng/ml (Fig. 1a). A higher concentration of FGF-2 (20 ng/ml) decreased the proliferation (data not shown). FGF-2-dependent cell growth was confirmed by observation with phase-contrast microscopy (Fig. 1b). FGF-2-mediated proliferation of hASCs was suppressed by specific inhibitor of FGFR (NVP-BGJ398, 0/0.05/0.1/1 μM) (Fig. 1c).

### FGF-2 promoted cell cycle transition from G0/G1 to S

**Fig. 2:**
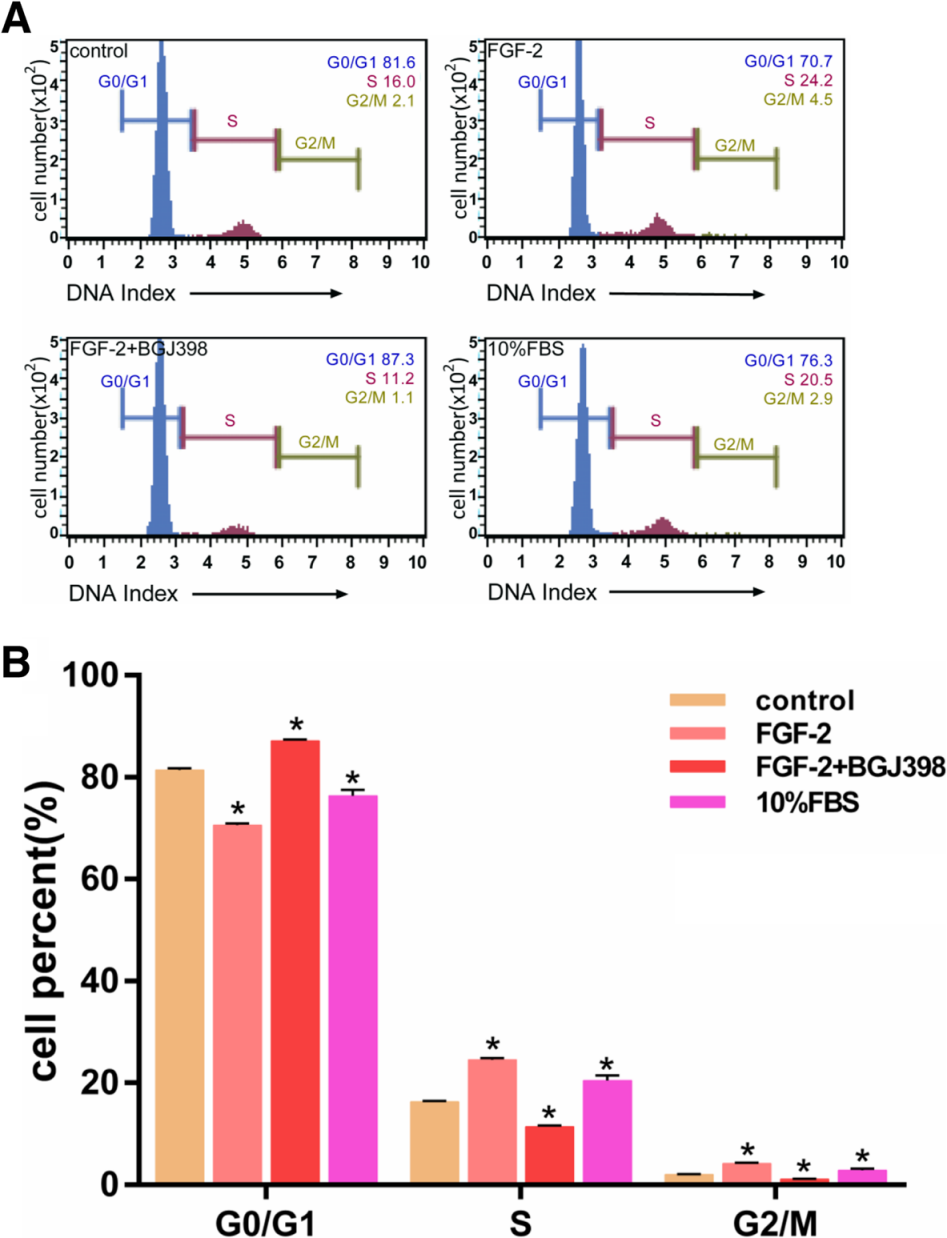
Analysis of the cell cycle in the effect of NVP-BGJ398 on FGF-2-mediated proliferation of hASCs. Cells were incubated with FGF-2 (5 ng/ml) with/without NVP-BGJ398 in serum-free DMEM for 48 h. Cell cycle stages determined by flow cytometry. **a** Cell cycle distributions in hASCs after treatment with FGF-2 with/without NVP-BGJ398 (0.1 μM) (*n* = 5). *p < 0.01 compared with controls. **b** Representative data from five independent experiments

When compared with the control group, flow cytometry in the FGF-2 group showed an increased trend in S and G2/M phases, and this phenomenon was inhibited in the FGF-2 with NVP-BGJ398 group (Fig. 2a). Namely, the G0/G1 phase increased with the inhibitor instead of the decrease of the S-phase. A histogram of the flow cytometry results is shown in Fig. 2b. The percentage of cells treated with FGF-2 in the S phase (24.56 ± 0.65%) was significantly higher than in controls (16.26 ± 0.47%). Similarly, the percentage of cells treated with FGF-2 in the G2/M phase (4.20 ± 0.32%) was also significantly higher compared with controls (2.02 ± 0.23%). Finally, the percentage of cells treated with FGF-2 with NVP-BGJ398 in the S and G2/M phases (11.4 ± 1.43% and 0.96 ± 0.34%, respectively) was also significantly lower compared with controls (16.26 ± 0.47% and 2.02 ± 0.23%, respectively).

### Signaling pathway protein kinase inhibitors suppress FGF-2-mediated proliferation of hASCs

**Fig. 3:**
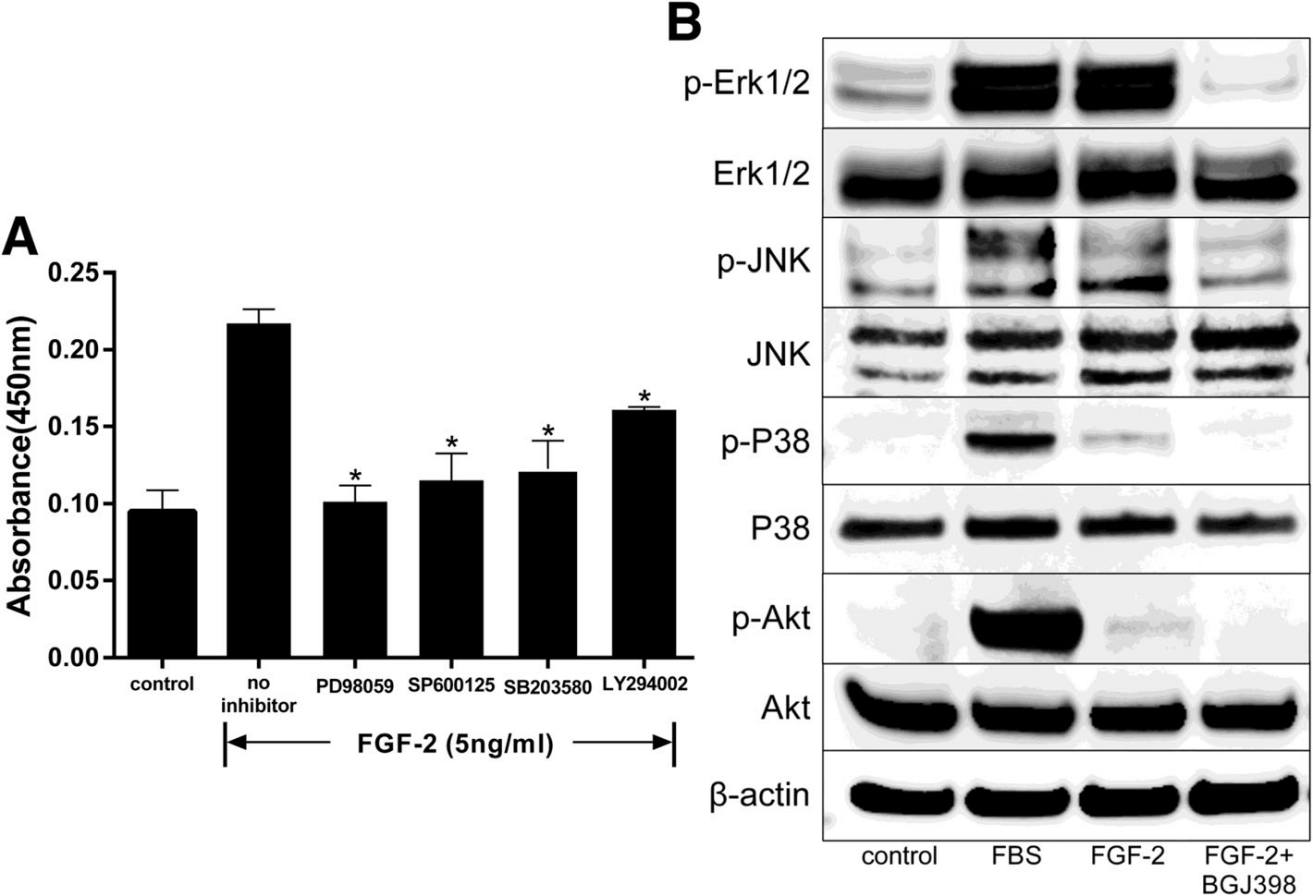
Stimulation of signal transduction in FGF-2-treated hASCs through multi-signaling pathways. After incubation in serum-free DMEM for 18 h, cells were treated with inhibitors at the designated concentrations [Erk1/2 inhibitor (PD98059, 5 μM), JNK inhibitor (SP600125, 10 μM), p38 MAPK inhibitor (SB203580, 20 μM), or PI3K/Akt inhibitor (LY294002, 10 μM)]. Cell proliferation was assessed with a Cell Counting Kit-8 after culturing with inhibitors for 48 h, and cellular proteins were extracted after cells were treated with FGF-2 with/without FGFR inhibitor NVP-BGJ398 (0.1 μM) for 5 min. Inhibitors were added 1 h prior to stimulation with FGF-2 (5 ng/ml). **a** Pharmacological inhibition of FGF-2-mediated proliferation through Erk1/2, JNK, p38 MAPK, and Akt pathways (*n* = 5). *p < 0.01 compared with no inhibitor. **b** Immunoblots of p-Akt, p-Erk1/2, p-p38 MAPK, and p-JNK in cells treated with FGF-2 with/without NVP-BGJ398

To examine the involvement of signaling pathways in the stimulation of hASCs by FGF-2, cells were treated with an Erk1/2 inhibitor (PD98059, 5 μM), a JNK inhibitor (SP600125, 10 μM), a p38 MAPK inhibitor (SB203580, 20 μM), or a PI3K/Akt inhibitor (LY294002, 10 μM). FGF-2-mediated cell proliferation was reduced by PD98059, SP600125, SB203580, and LY294002 (Fig. 3a).

We also examined which protein kinases of signaling pathways were activated in the stimulation of hASCs by FGF-2. hASCs were treated with FGF-2 with/without NVP-BGJ398, followed by analysis by immuno-blotting. Phosphorylation of Erk, JNK, p38, and Akt was increased in FGF-2-treated cells. However, NVP-BGJ398 inhibited the phosphorylation of these enzymes even in the presence of FGF-2 (Fig. 3b).

### FGF-2 stimulated proliferation through Src activation in hASCs

**Fig. 4:**
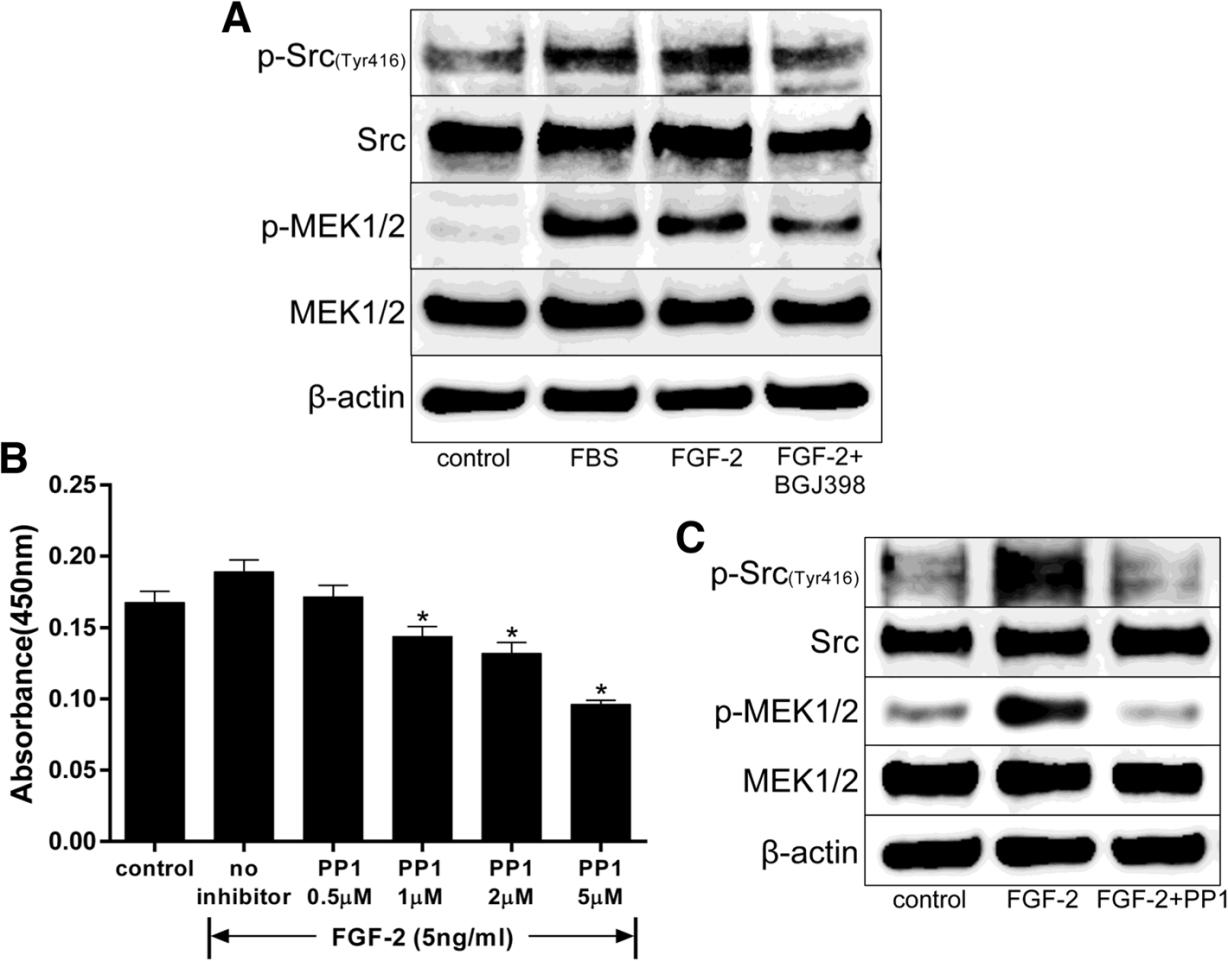
Src activation in FGF-2-treated hASCs. After incubation in serum-free DMEM for 18 h, cells were treated with FGF-2 with/without NVP-BGJ398 (0.1 μM) or PP1 (5 μM). Cell proliferation was assessed with a Cell Counting Kit-8 after culturing for 48 h. The cell lysates were prepared after treatment for 5 min. NVP-BGJ398 or PP1 was added 1 h prior to stimulation with FGF-2 (5 ng/ml). **a** Immunoblots of p-Src (Tyr416) and p-MEK1/2 under the treatment of cells with FGF-2 with/without NVP-BGJ398. **b** Effect of PP1 (Src selective inhibitor) (0/0.5/1/2/5 μM) on FGF-2-dependent proliferation of hASCs (*n* = 6). *p < 0.01 compared with no inhibitor. **c** Immunoblots of p-Src (Tyr416) and p-MEK1/2 in cells treated with FGF-2 with/without PP1

To clarify the involvement of signaling pathways downstream of FGFR and upstream of Erk1/2 in the stimulation of hASCs by FGF-2, activation of Src was analyzed. The level of p-Src was increased by FGF-2-mediated proliferation, and NVP-BGJ398 inhibited this phosphorylation even in the presence of FGF-2 (Fig. 4a). MEK1/2, an enzyme downstream of Src, was also phosphorylated by FGF-2, but was not activated by NVP-BGJ398 treatment. MEK1/2, an enzyme downstream of Src, was also phosphorylated by FGF-2, but was not activated by NVP-BGJ398 treatment. When hASCs were also treated with a Src-selective inhibitor (PP1, 0/0.5/1/2/5 μM), FGF-2-mediated cell proliferation was suppressed (Fig. 4b). Additionally, PP1 inhibited the phosphorylation of Src and MEK1/2 even in the presence of FGF-2 (Fig. 4c). Thus, FGF-2 stimulated cell proliferation through Src activation, followed by mediation by multiple signaling pathways.

## Discussion

Our results showed that proliferation of hASCs was enhanced by FGF-2 when the concentration was lower than 10 ng/ml, while an FGFR inhibitor and various signaling pathway inhibitors (Erk1/2 inhibitor, PI3K/Akt inhibitor, JNK inhibitor, p38 MAPK inhibitor, and Src inhibitor) inhibited FGF-2-mediated proliferation. There was also found to be a greater number of cells in S and G2/M phases in the FGF-2 group compared with the control group, and an FGFR inhibitor canceled this effect. The levels of p-Erk1/2, p-JNK, p-p38, p-Akt, p-Src, and p-MEK1/2 were decreased by an FGFR inhibitor, while p-Src and p-MEK1/2 were decreased by a Src inhibitor, indicating that FGF-2 enhanced proliferation of hASCs through multiple signaling pathways by activating Erk1/2, JNK, p38, Akt, Src, and MEK1/2.

hASCs, a subset of mesenchymal stem cells (MSCs), were first reported to be multipotent stem cells nearly 20 years ago, and have since been regarded as one of the most promising stem cells [2]. The beneficial characteristics of stem cells are their ability to renew their own populations and to differentiate into multiple cell lineages, and thus, they have been defined as units of biological organization responsible for the development and regeneration of organ and tissue systems. At present, there are four main sources of stem cells: embryonic tissues, fetal tissues, adult tissues, and induced pluripotent stem cells (iPSCs) [32]. Stem cells can be differentiated into the desired phenotype when seeded in an appropriate composition in vitro and can be applied directly into areas of damaged tissues and organs in situ [8]. Adult stem cells can be harvested from almost every part of the body in adult organisms, including from fat, skin, bone marrow, blood, or skeletal muscle. While only a limited amount of cells can be harvested, the use of hASCs is popular due to their potential for advanced tissue engineering and cell therapies, as well as due to their ubiquity and ease of harvest [3, 4], later senescence [5, 6], higher proliferation capacity [7], greater activity in autocrine production [8, 9], less ethical and legal issues [8, 10], and ability to be cryopreserved for a long time [11]. Despite the advantages of hASCs, including their stable gene expression, their differentiation potential and proliferation rate begins to slow down significantly with time, and addressing how to maintain the abilities of these stem cells is an important issue for basic and clinical study [20, 33]. As reported by Agostini et al. [34], hASC did not show altered phenotype and genetic lesion after expansion between P12 (12 passages) and P14 when compared with P1 [35]; although the proliferation rate slows down after P15, alterations of biological characteristics are not observed [33]. In this study, we used hASC in P7-P9 which keep proliferation ability. In our previously study, we found that hypoxia, FGF-2, TGF-β1, PRP, and PDGF-BB can induce the proliferation and differentiation of hASC [22, 36, 37]. Zaragosi et al. found that FGF-2 plays a crucial role in self-renewal and can restore differentiation and proliferation abilities in hASCs [20]. Thus, further research on the effect of FGF-2 on hASCs is warranted.

**Fig. 5:**
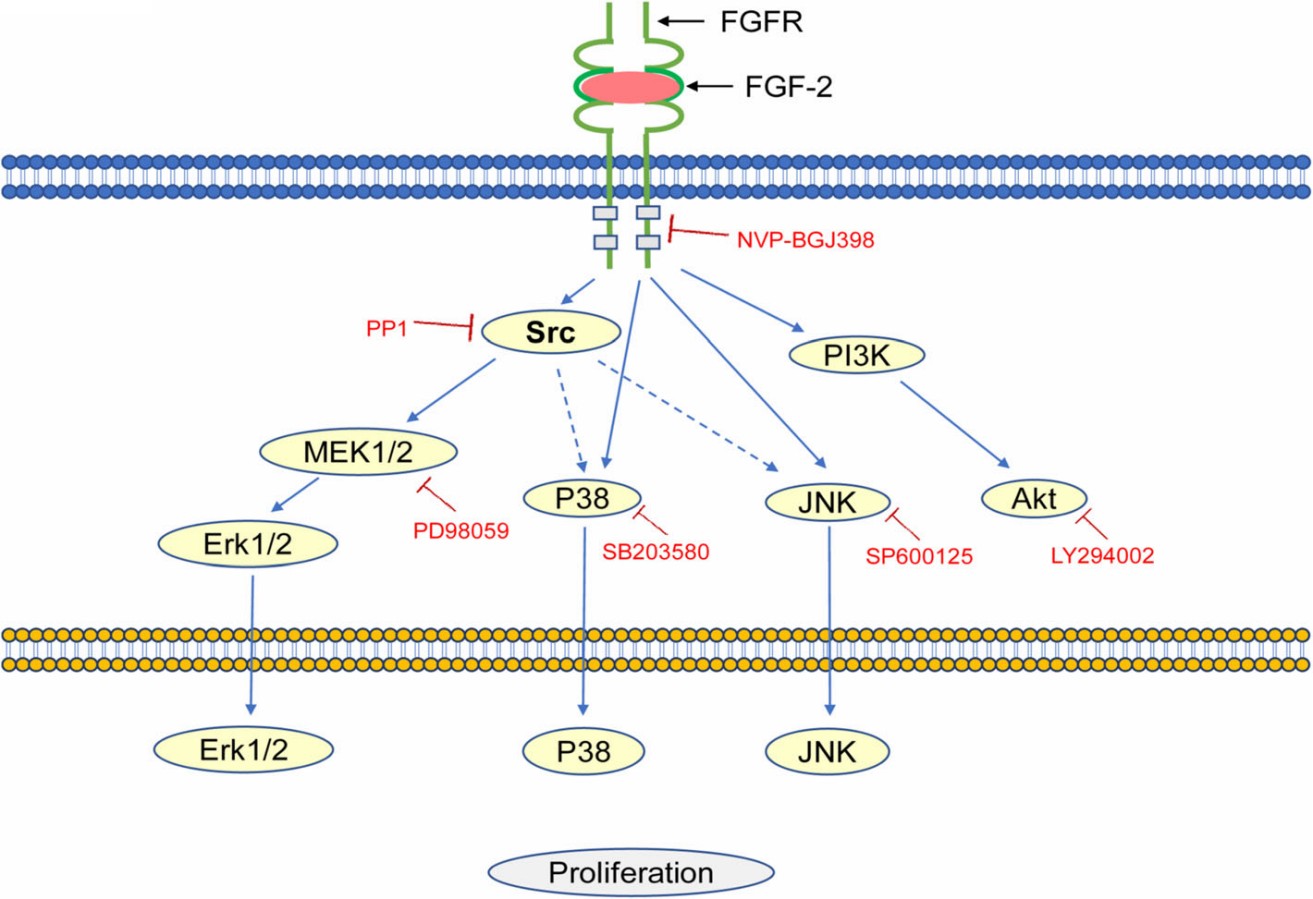
Possible FGF-2-signal pathways. NVP-BGJ398, PP1, PD98059, SB203580, SP600125, and LY294002 are specific inhibitors of FGFR, Src, MEK1/2, p38 MAP kinase, JNK, and Akt, respectively

FGF-2 is a heparin-binding protein belonging to the FGF family that is distributed among tissues and combined with proteoglycan of the cell surface. It is known that the signaling component of the mammalian FGF family is comprised of 18 secreted proteins that interact with four signaling tyrosine kinase FGF receptors (FGFRs) and a class of low affinity receptors, the heparan sulfate proteoglycans (HSPGs). Interaction of FGF ligands with their signaling receptors is regulated by protein or proteoglycan cofactors, and via extracellular binding proteins, activated FGFRs phosphorylate specific tyrosine residues that mediate the interaction with cytosolic adaptor proteins and RAS-MAPK, PI3K-AKT, PLCγ, and STAT intracellular signaling pathways [38]. These phosphorylated tyrosine residues are recognized by SH2 domain-containing signal transducers, and target enzymes such as PLCγ and Src bind to the tyrosine autophosphorylation sites and themselves become phosphorylated (Fig. 5). The role of kinases such as Src, PKC, or P13-kinase seems to depend on the cell type. The FGF-2 pathway is one of the most significant regulators of proliferation of human embryonic stem cells (hESCs) and oncogenesis of tumors [39], whereas FGF signaling is important in early vertebrate development through the Ras/MAPK pathway, PLCγ/Ca^2+^pathway, and PI3 kinase/Akt pathway [40]. In fact, FGF-2 activated the PLCγ-PKC pathway as well as Src in endothelial cells, and the differentiation was independent of PKC signals [41]. Since we did not examine whether the PLCγ/Ca^2+^-PKC pathway in hASCs is activated by FGF-2, the involvement of PKC in the proliferation and differentiation of hASCs is unclear. The present study clearly showed that inhibition of Src strongly reduced the proliferation of hASCs, thereby indicating that Src plays an important role in their maintenance.

FGF-2 is clinically approved in Japan and included in insurance [42]. Our previous studies show FGF-2 can induce the proliferation and differentiation of hASCs [22, 43]; however, the signaling pathways involved have remained unclear. In the presented study, we revealed that the most effective concentration of FGF-2 for proliferation of hASCs was 5 ng/ml, which is about 1000 times the FGF-2 concentration in plasma (< 6.4 ng/l) and serum (< 4.0 ng/l in men, < 10.8 ng/l in women) [44]. FGF-2 stimulated cell progression to the S and G2/M phases of the cell cycle, and an FGFR inhibitor decreased this effect, indicating that FGF-2 can maintain hASC proliferation ability in vitro. Activation of Erk1/2, JNK, p38, Akt, and especially the Erk1/2 pathway may play a crucial role in FGF-2-mediated hASC proliferation, which is consistent with the findings of Zaragosi et al. [20]. Mitogen-activated protein kinases (MAPKs), including Erk1/2, JNK, and p38, are known to regulate diverse cellular programs including embryogenesis, proliferation, differentiation, and apoptosis [45]. In a variety of cells, Erk1/2, JNK, and p38 MAPK activation are associated with cell proliferation, differentiation, and survival [46], while Akt activation is associated with survival [47]. Our data show that FGF-2 can enhance the proliferation in hASCs by activation of Erk1/2, JNK, p38 MAPK, and Akt, especially in the Erk1/2 pathway, and that FGF-2 can maintain the proliferation, differentiation, and survival of hASCs. In contrast, Lee et al. found that phosphorylation of p38, Erk, and JNK was decreased, while the phosphorylation of Src kinase was increased, by treatment of hASCs with FGF-2 and dexamethasone (DEX) [48]. This difference may be explained by the effect of DEX and requires further investigation.

Src is an Src kinase family member and is anchored to the cytoplasmic side of the cell membrane [49]. PP1 (4-Amino-5-(4-methylphenyl)-7-(t-butyl) pyrazolo [3,4-d]-pyrimidine) was reported as a Src-selective tyrosine kinase inhibitor and has been used extensively to investigate signaling pathways involving Src kinases, including events downstream of the stem cell factor (SCF) receptor [50]. Recent studies show that in some malignancies and arthritis, Src kinase activity is upregulated, which induces cell migration and invasion [50–52]. Additionally, Src inhibition is an important anti-tumor therapy [53]. A previous study showed that Src is activated by FGF-2 and DEX in hASCs [48]. The present study showed that inhibitors for various signal transduction enzymes, and an FGFR inhibitor, blocked FGF-2-mediated proliferation. Phosphorylation of Src and MEK1/2 increased after FGF-2 treatment and decreased after administration of the FGFR inhibitor NVP-BGJ398 and the Src inhibitor PP1. These results indicate that activation of MEK1/2, a downstream enzyme of Src, is necessary for FGF-2-mediated proliferation (Fig. 5). PP1 can inhibit the Src kinase family members Lck, FynT, Hck, and Src [54], and further studies are needed to clarify the details of these signal transductions. Our data show that FGF-2 can enhance Src/MEK1/2 activity in hASCs, followed by the phosphorylation of multiple signals including Erk1/2, p38MAPK, and JNK (Fig. 5). p-Akt was also independently elevated by FGF-2. In conclusion, hASC proliferation and differentiation are under the influence of FGF-2; however, it is difficult to know whether the same cell uses FGF-2 to generate two different pathways within itself that lead to both proliferation and migration, or whether a cell generates a single intracellular pathway able to trigger both responses [55]. Based on the function of Src in the migration and invasion of cancer cells, FGF-2 may increase the migration ability as well as the proliferation ability of hASCs, and we will continue to conduct further research on the safety and utility of hASCs for clinical application.

## Conclusions

FGF-2 at concentrations lower than 10 ng/ml enhanced hASC proliferation. FGF-2 enhancement of hASC proliferation was mediated by several signaling pathways. Src activation is essential for the activation of multiple downstream pathways in FGF-2-mediated proliferation of hASCs.

## Data Availability

All data generated and/or analyzed during this study are included in this published article.

## Abbreviations

Akt: Ak strain transforming
Erk: Extracellular-signal-regulated kinase
JNK: c-Jun N-terminal kinase
MEK: Mitogen-activated protein kinase kinase
p38: p38 mitogen-activated protein kinases
Src: Proto-oncogene tyrosine-protein kinase Src

## References

[1] Zuk PA (2001). Multilineage cells from human adipose tissue: implications for cell-based therapies. Tissue Eng.

[2] Zuk P (2013). Adipose-derived stem cells in tissue regeneration: a review. ISRN Stem Cells.

[3] Tobita M, Orbay H, Mizuno H (2011). Adipose-derived stem cells: current findings and future perspectives. Discov Med.

[4] Frolich K, Hagen R, Kleinsasser N (2014). Adipose-derived stromal cells (ASC) - basics and therapeutic approaches in otorhinolaryngology. Laryngorhinootologie.

[5] Ding DC (2013). Human adipose-derived stem cells cultured in keratinocyte serum free medium: Donor's age does not affect the proliferation and differentiation capacities. J Biomed Sci.

[6] Kokai LE, Marra K, Rubin JP (2014). Adipose stem cells: biology and clinical applications for tissue repair and regeneration. Transl Res.

[7] Barba M (2013). Adipose-derived mesenchymal cells for bone regereneration: state of the art. Biomed Res Int.

[8] Bacakova L (2018). Stem cells: their source, potency and use in regenerative therapies with focus on adipose-derived stem cells - a review. Biotechnol Adv.

[9] Vizoso FJ, et al. Mesenchymal stem cell secretome: toward cell-free therapeutic strategies in regenerative medicine. Int J Mol Sci. 2017;18(9):1852. 10.3390/ijms18091852 PMC5618501 28841158

[10] Palumbo P, et al. Methods of isolation, characterization and expansion of human adipose-derived stem cells (ASCs): an overview. Int J Mol Sci. 2018;19(7):1897. 10.3390/ijms19071897 PMC6073397 29958391

[11] Devitt SM (2015). Successful isolation of viable adipose-derived stem cells from human adipose tissue subject to long-term cryopreservation: positive implications for adult stem cell-based therapeutics in patients of advanced age. Stem Cells Int.

[12] Louwen F (2018). Insight into the development of obesity: functional alterations of adipose-derived mesenchymal stem cells. Obes Rev.

[13] Raposio E, Bertozzi N (2017). Isolation of ready-to-use adipose-derived stem cell (ASC) pellet for clinical applications and a comparative overview of alternate methods for ASC isolation. Curr Protoc Stem Cell Biol.

[14] Mitsiadis TA, Orsini G (2016). Editorial: a new era in dentistry: stem cell-based approaches for tooth and periodontal tissue regeneration. Front Physiol.

[15] Ornitz DM, Itoh N (2001). Fibroblast growth factors. Genome Biol.

[16] Nandy SB, et al. Fibroblast growth factor-2 alone as an efficient inducer for differentiation of human bone marrow mesenchymal stem cells into dopaminergic neurons. J Biomed Sci. 2014;21:83. 10.1186/s12929-014-0083-1 PMC4190371 25248378

[17] Hu Y (2016). Effects of nerve growth factor and basic fibroblast growth factor dual gene modification on rat bone marrow mesenchymal stem cell differentiation into neuron-like cells in vitro. Mol Med Rep.

[18] Pizzute T (2016). Fibroblast growth factor ligand dependent proliferation and chondrogenic differentiation of synovium-derived stem cells and concomitant adaptation of Wnt/mitogen-activated protein kinase signals. Tissue Eng Part A.

[19] Rider DA (2008). Autocrine fibroblast growth factor 2 increases the multipotentiality of human adipose-derived mesenchymal stem cells. Stem Cells.

[20] Zaragosi LE, Ailhaud G, Dani C (2006). Autocrine fibroblast growth factor 2 signaling is critical for self-renewal of human multipotent adipose-derived stem cells. Stem Cells.

[21] Hebert TL (2009). Culture effects of epidermal growth factor (EGF) and basic fibroblast growth factor (bFGF) on cryopreserved human adipose-derived stromal/stem cell proliferation and adipogenesis. J Tissue Eng Regen Med.

[22] Kakudo N (2015). Hypoxia enhances proliferation of human adipose-derived stem cells via HIF-1a activation. PLoS One.

[23] Thomas SM, Brugge JS (1997). Cellular functions regulated by Src family kinases. Annu Rev Cell Dev Biol.

[24] Dexter TM, Boettiger D, Spooncer E (1985). Self-renewal of haemopoietic stem cells: the roles of the environment, of growth factors and of the src oncogene. Haematol Blood Transfus.

[25] Orschell CM (2008). Deficiency of Src family kinases compromises the repopulating ability of hematopoietic stem cells. Exp Hematol.

[26] Meyn MA, Smithgall TE. Chemical genetics identifies c-Src as an activator of primitive ectoderm formation in murine embryonic stem cells. Sci Signal. 2009;2(92):ra64–ra64. 10.1126/scisignal.2000311 PMC2775445 19825829

[27] Chen JX (2011). Involvement of c-Src/STAT3 signal in EGF-induced proliferation of rat spermatogonial stem cells. Mol Cell Biochem.

[28] Chetty S (2015). A Src inhibitor regulates the cell cycle of human pluripotent stem cells and improves directed differentiation. J Cell Biol.

[29] Zhang X (2014). Src-family tyrosine kinase activities are essential for differentiation of human embryonic stem cells. Stem Cell Res.

[30] Kakudo N (2008). Proliferation-promoting effect of platelet-rich plasma on human adipose-derived stem cells and human dermal fibroblasts. Plast Reconstr Surg.

[31] Zuk PA (2002). Human adipose tissue is a source of multipotent stem cells. Mol Biol Cell.

[32] Si Z (2019). Adipose-derived stem cells: sources, potency, and implications for regenerative therapies. Biomed Pharmacother.

[33] Li J, Huang H, Xu X (2017). Biological characteristics and karyotiping of a new isolation method for human adipose mesenchymal stem cells in vitro. Tissue Cell.

[34] Agostini F (2018). Improved GMP compliant approach to manipulate lipoaspirates, to cryopreserve stromal vascular fraction, and to expand adipose stem cells in xeno-free media. Stem Cell Res Ther.

[35] Neri S (2013). Human adipose stromal cells (ASC) for the regeneration of injured cartilage display genetic stability after in vitro culture expansion. PLoS One.

[36] Lai F (2018). Platelet-rich plasma enhances the proliferation of human adipose stem cells through multiple signaling pathways. Stem Cell Res Ther.

[37] Kakudo N (2012). Effects of transforming growth factor-beta1 on cell motility, collagen gel contraction, myofibroblastic differentiation, and extracellular matrix expression of human adipose-derived stem cell. Hum Cell.

[38] Ornitz DM, Itoh N (2015). The fibroblast growth factor signaling pathway. Wiley Interdiscip Rev Dev Biol.

[39] Dvorak P, Dvorakova D, Hampl A (2006). Fibroblast growth factor signaling in embryonic and cancer stem cells. FEBS Lett.

[40] Bottcher RT, Niehrs C (2005). Fibroblast growth factor signaling during early vertebrate development. Endocr Rev.

[41] Klint P (1999). Contribution of Src and Ras pathways in FGF-2 induced endothelial cell differentiation. Oncogene.

[42] Hakuba N (2003). A new method for closing tympanic membrane perforations using basic fibroblast growth factor. Laryngoscope.

[43] Kakudo N, Shimotsuma A, Kusumoto K (2007). Fibroblast growth factor-2 stimulates adipogenic differentiation of human adipose-derived stem cells. Biochem Biophys Res Commun.

[44] Larsson A, Skoldenberg E, Ericson H (2002). Serum and plasma levels of FGF-2 and VEGF in healthy blood donors. Angiogenesis.

[45] Raman M, Chen W, Cobb MH (2007). Differential regulation and properties of MAPKs. Oncogene.

[46] Johnson GL, Lapadat R (2002). Mitogen-activated protein kinase pathways mediated by ERK, JNK, and p38 protein kinases. Science.

[47] Fischer U, Janicke RU, Schulze-Osthoff K (2003). Many cuts to ruin: a comprehensive update of caspase substrates. Cell Death Differ.

[48] Lee SY (2009). Enhanced ex vivo expansion of human adipose tissue-derived mesenchymal stromal cells by fibroblast growth Factor-2 and dexamethasone. Tissue Eng A.

[49] Karni R, Levitzki A (2000). pp60(cSrc) is a caspase-3 substrate and Is essential for the transformed phenotype of A431 cells. Mol Cell Biol Res Commun.

[50] Summy JM, Gallick GE (2003). Src family kinases in tumor progression and metastasis. Cancer Metastasis Rev.

[51] Krishnan H (2019). Src and podoplanin forge a path to destruction. Drug Discov Today.

[52] Tatton L (2003). The Src-selective kinase inhibitor PP1 also inhibits Kit and Bcr-Abl tyrosine kinases. J Biol Chem.

[53] Gnoni A (2011). Dasatinib: an anti-tumour agent via Src inhibition. Curr Drug Targets.

[54] Hanke JH (1996). Discovery of a novel, potent, and Src family-selective tyrosine kinase inhibitor. Study of Lck- and FynT-dependent T cell activation. J Biol Chem.

[55] Boilly B (2000). FGF signals for cell proliferation and migration through different pathways. Cytokine Growth Factor Rev.

